# Controlled Synthesis of Copper Sulfide Nanoparticles in Oxygen‐Deficient Conditions Using Flame Spray Pyrolysis (FSP) and Its Potential Application

**DOI:** 10.1002/smll.202409993

**Published:** 2025-03-09

**Authors:** Muhammad Ali Martuza, Suman Pokhrel, Jakob Stahl, Marco Schowalter, Andreas Rosenauer, Lutz Mädler

**Affiliations:** ^1^ Faculty of Production Engineering University of Bremen Badgasteiner Straße 1 28359 Bremen Germany; ^2^ Process Engineering Department Leibniz Institute for Materials Engineering IWT Badgasteiner Straße 3 28359 Bremen Germany; ^3^ MAPEX Center for Materials and Processes University of Bremen D‐28359 Bremen Germany; ^4^ Institute of Solid State Physics University of Bremen Otto‐Hahn‐Allee 1 28359 Bremen Germany

**Keywords:** bandgap, Co‐flow rate, flame spray pyrolysis, fuel‐to‐oxygen ratio, metal sulfide, metal‐to‐sulfur ratios, precursor flow

## Abstract

The objective of this study is to investigate the influence of various process parameters, such as the fuel‐to‐oxygen ratio, precursor flow rate, co‐flow rate, and different metal‐to‐sulfur ratios on the properties of metal sulfide particles synthesized via flame spray pyrolysis (FSP). The particle size increases with increasing dispersion oxygen flow and copper sulfide is obtained only when the fuel‐to‐oxygen ratio is equal to or higher than 1.5. The temperature of the flame rises with an increasing precursor flow rate and copper sulfide is formed at a precursor flow rate of 5 mL min^−1^ or lower, while contamination occurs above 5 mL min^−1^. A Co‐flow rate above 100 L min^−1^ is required to cool the aerosol stream before deposition on the filter. A pure copper sulfide phase is produced when sulfur is more than 5 times in molar ratio compared to Cu in the liquid solution and particle size decreases with increasing sulfur concentration. This research will contribute to a better understanding of the fundamental formation process of metal sulfides under oxygen‐lean gas‐phase conditions and serve as a milestone in optimizing synthesis parameters for various applications.

## Graphical abstract

This study investigates how process parameters such as precursor‐solvent interaction, fuel‐to‐oxygen ratio, precursor flow rate, co‐flow rate, and metal‐to‐sulfur ratios affect flame spray pyrolysis (FSP)‐synthesized metal sulfide properties. Based on optical bandgap and electrochemical characterization, it is predicted that copper sulfide has potential applications in the field of gas sensors, photo‐catalysts, and solar cells.

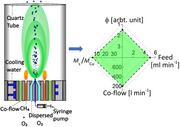

## 1 Introduction

FSP is demonstrated as an established technology for the one‐step synthesis of single‐metal, multi‐metal oxide nanoparticles,^[^ ^1^ ^]^ applicable at both laboratory and industrial production scales.^[^ ^2^ ^]^ The key factor behind the remarkable success of FSP is its convenient process of precursor‐solvent preparation by dissolving metal‐containing precursor in an organic solution, which can be used for almost all elements in the periodic table.^[^ ^3^ ^]^ FSP‐synthesized high‐purity products exhibit unique morphologies with high‐temperature crystal phases that are metastable under ambient conditions,^[^ ^4^ ^]^ resulting in diverse applications in gas sensors,^[^ ^5^ ^]^ water splitting,^[^ ^6^ ^]^ catalysts,^[^ ^5^, ^7^ ^]^ photonics,^[^ ^8^ ^]^ health care,^[^ ^9^ ^]^ magnetic materials,^[^ ^10^ ^]^ fuel cells and batteries,^[^ ^5^, ^11^ ^]^ bio‐imaging,^[^ ^12^ ^]^ and nano‐toxicology studies.^[^ ^13^ ^]^ The FSP process also enables in situ mixing,^[^ ^14^ ^]^ coating,^[^ ^15^ ^]^ doping,^[^ ^13^, ^16^ ^]^ and functionalization^[^ ^17^ ^]^ of the nanoparticle along with a continuous production process by simply optimizing the process parameters which makes it a cost‐efficient and attractive choice for large‐scale industrial production. However, despite its dominant capabilities and advantages, FSP has primarily been confined to metal oxides, creating a strong demand to expand its applications to metal sulfides.

In a typical FSP process, a liquid solution is prepared by dissolving a metal‐organic precursor in a highly combustible organic solvent. The solution is then atomized with dispersed oxygen using a high‐pressure nozzle to form a spray of micrometer‐sized droplets. The spray is ignited by a premixed flame and subsequently, droplets undergo micro‐explosions due to the superheating of highly volatile components trapped inside them. The metal components within the droplets absorb heat from the flame and evaporate into vapor. The vapor then undergoes gas phase thermal decomposition and nucleation begins after supersaturation of the metal precursor vapor. This nucleation is then followed by particle growth, coalescence, agglomeration, and sintering. This typical open flame reactor is suitable for metal oxide formation, where nanoparticles are synthesized through the oxidization of metal precursors but not suitable for metal sulfide production, which requires a reducing reaction environment. The modification of the open flame reactor with an enclosed flame reactor enables precise control over the fuel‐to‐oxygen ratio, a critical factor for synthesizing metal sulfide nanoparticles.^[^ ^18^ ^]^ This enclosed reactor also effectively prevents unwanted air entrainment into the flame, which helps to control the flame temperatures and particle residence time and, in turn, leads to better control over particle size,^[^ ^19^ ^]^ phase composition,^[^ ^20^ ^]^ phase purity, and crystallinity of the nanoparticles.^[^ ^10^, ^21^ ^]^ Even though metal sulfide synthesis using the FSP setup requires challenging process conditions, Pokhrel et al.^[^ ^18^ ^]^ successfully synthesized eight metal sulfides (MnS, CoS, Cu_2_S, ZnS, Ag_2_S, In_2_S_3_, SnS, Bi_2_S_3_) by carefully controlling the oxygen supply to the flame and maintaining a sufficient amount of metal‐to‐sulfur ratio. However, the fundamental understanding of the metal sulfide formation mechanisms in the gas phase and the impact of process parameters on the particle characteristics remain largely unexplored.

Among metal sulfides, copper sulfide is an important p‐type semiconductor with significant potential for applications in sensors,^[^ ^22^ ^]^ photocatalysts,^[^ ^23^ ^]^ cathode materials for lithium batteries,^[^ ^24^ ^]^ and solar cells.^[^ ^25^ ^]^ So far reported synthesis methods for copper sulfide are hydrothermal,^[^ ^26^ ^]^ solvothermal,^[^ ^27^ ^]^ thermolysis,^[^ ^28^ ^]^ microemulsion,^[^ ^29^ ^]^ solid‐state reaction,^[^ ^30^ ^]^ chemical vapor deposition,^[^ ^31^ ^]^ wet chemical method,^[^ ^32^ ^]^ and poly route^[^ ^33^ ^]^ resulting in various morphologies such as nanowires,^[26b]^ nanodisks,^[^ ^34^ ^]^ flower‐like structures,^[^ ^35^ ^]^ and hollow spheres.^[^ ^36^ ^]^ However, most of these approaches are incompatible with large‐scale production because of their expensive post‐processing and low production efficiency. In contrast, the FSP method has demonstrated a promising approach in synthesizing metal oxides at high‐volume production,^[^ ^2^ ^]^ and with a thorough understanding of process conditions, this capability can be extended to metal sulfides.

The main objective of this study is to investigate the precursor‐solvent chemistry and the impact of process parameters (feed rate, fuel‐to‐oxygen ratio, metal‐to‐sulfur molar ratio, and co‐flow) on the properties of metal sulfide nanoparticles synthesized via FSP. For this purpose, copper sulfide synthesis was studied and the corresponding precursor solution (Cu 2‐ethylhexanoate (Cu 2‐EHA), tetrahydrofuran (THF), and thiophene solution) was analyzed using Fourier Transform Infrared Spectroscopy (FTIR) and UV–vis spectroscopy. The produced copper sulfide nanoparticles were characterized using X‐ray diffraction (XRD), Transmission Electron Microscopy (TEM), Energy‐Dispersive X‐ray (EDX), and Brunauer–Emmelt–Teller (BET) techniques. UV–vis and Electrochemical Impedance Spectroscopy (EIS) were performed for copper sulfide particles to determine the bandgap and flat band potential respectively.

## 2 Results and Discussion

### 2.1 Precursor‐Solvent Analysis

Infrared spectroscopy (IR) provides valuable information about molecular vibration and functional groups present in a solution and is highly sensitive to the breakage and formation of covalent bonds. A systematic analysis of precursor‐solvent combinations was conducted using IR spectroscopy to understand solution stability and the influence of precursor‐solvent chemistry on particle formation. Moreover, UV–vis spectroscopy provides insights into the electronic structure and bonding interactions of metal‐ligand complexes. Among various sulfur sources,^[^ ^37^ ^]^ the experiment in this study used thiophene as the sulfur source, Cu‐2‐ethylhexanoate as the metal precursor, and tetrahydrofuran as the solvent to enhance the solubility of both the metal precursor and the sulfur source.

**Figure 1:**
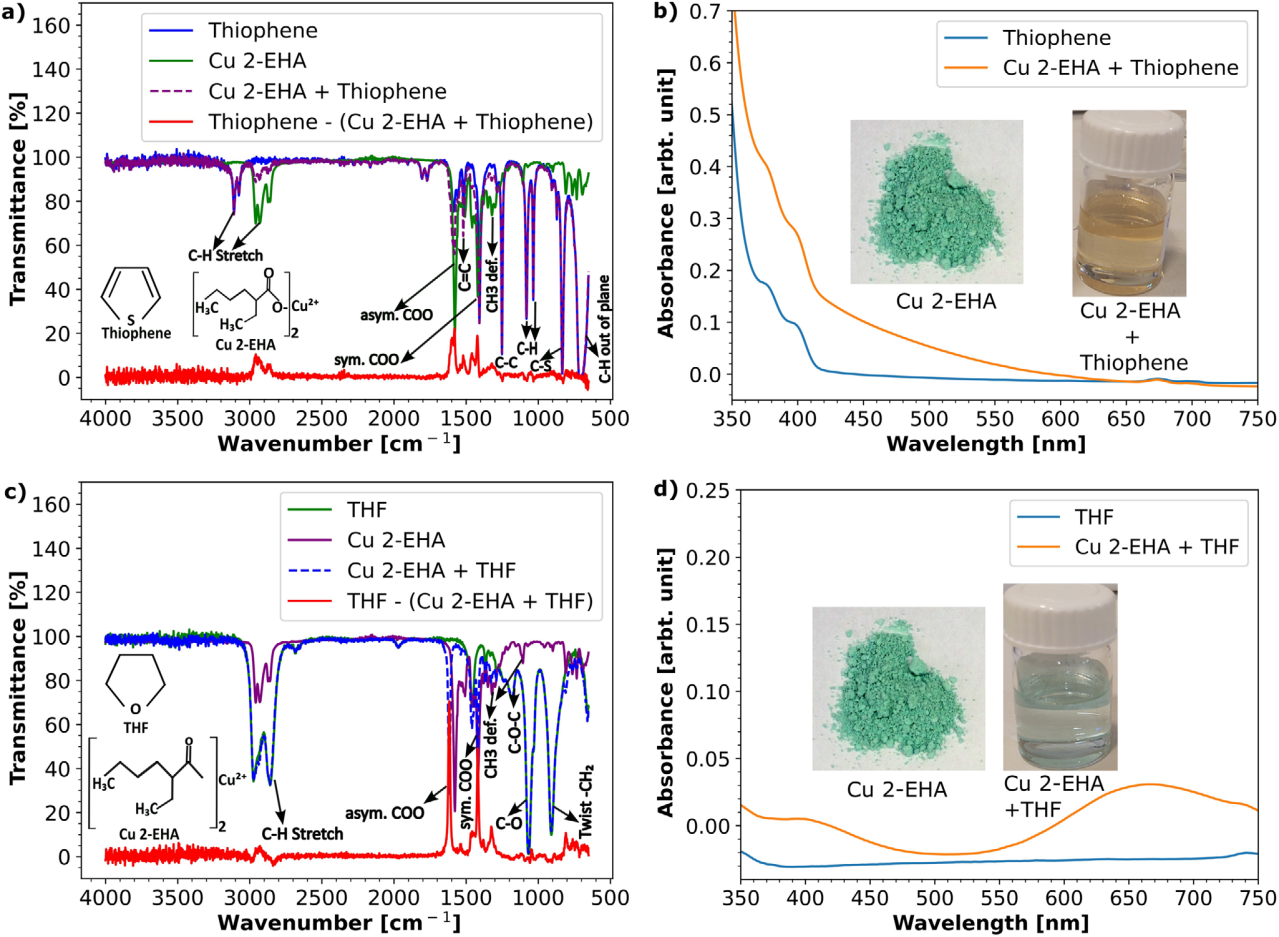
a) IR spectra of Cu 2‐EHA show C─H symmetric and asymmetric vibration at ≈(2958–2925) cm^−1^, asymmetric and symmetric COO^−^ vibration at 1576 and 1416 cm^−1^, and ─CH_3_ deformation peaks at ≈1319 and 1105 cm^−1^. The IR spectra of thiophene show the typical characteristic thiophene ring deformation at 1407 cm^−1^, symmetric and asymmetric vibration of C═C at ≈1587 and 1518 cm^−1^, C─C vibration at 1250 cm^−1^, in‐plane stretching vibrations of C─H at ≈3109, 1033, and 1081 cm^−1^, out‐of‐plane C─H bending vibrations at ≈(712–695) cm^−1^, and C─S at 833 cm^−1^. The IR spectra of Cu 2‐EHA and thiophene solution are shown with a dashed‘—‘line. The red line indicates the difference spectra of thiophene and the solution of Cu 2‐EHA and thiophene. b) UV–vis spectroscopy of pure thiophene (blue line) and the solution of Cu 2‐EHA and thiophene (yellow line). Thiophene is a transparent solution but after mixing with Cu 2‐EHA it becomes a pale yellow color as shown in a photograph. C) IR spectra of THF show symmetric and asymmetric C─H stretch vibrations at 2972 cm^−1^ and 2858 cm^−1^, deformation, wag, and twist of ─CH_2_ compound respectively 1460 cm^−1^, 1365 cm^−1^, 908 cm^−1^, and C─O─C asymmetric stretch vibration at 1181 cm^−1^, C─O bond at 1065 cm^−1^. The IR spectra of Cu 2‐EHA and THF solution are shown by a dashed ‘—‘line. The red line indicates the difference spectra of THF and the solution of Cu 2‐EHA and THF. d) UV–vis spectroscopy of pure THF, and a solution of Cu 2‐EHA and THF. After mixing Cu 2‐EHA with THF, Cu 2‐EHA retains its original color in the solution as shown in the photograph.

**Figure** **1**a shows a decrease in the transmittance intensity of the C═C stretching vibration of thiophene at 1518 cm^−1^, while the intensity of other bonds remains unchanged when thiophene is in a solution with Cu 2‐EHA. This indicates that only the nature of the C═C bond is affected. This change can be interpreted as a consequence of a new coordination bond with the C═C bond of thiophene. Moreover, the asymmetric and symmetric COO^−^ stretch bonding signals of the Cu 2‐EHA appear at ν_a_ = 1576 cm^−1^ and ν_s_ = 1417cm^−1^, respectively (Δν = ν_a_ – ν_s_ = 159 cm^−1^) while the same peaks in the Cu 2‐EHA and thiophene solution shift to ν_a_ = 1581 cm^−1^ and ν_s_ = 1407 cm^−1^, respectively (Δν = ν_a_ – ν_s_ = 174 cm^−1^). The position of the fundamental COO^−^ stretching vibration and the value of their difference Δν indicate a change in the coordination mode of the metal ion with COO^−^.^[^ ^38^ ^]^ The difference of the asymmetric and symmetric COO^−^ stretching vibration increases in the Cu 2‐EHA and thiophene solution compared to solid Cu 2‐EHA, which implies that COO^−^ becomes more ionic in nature in the solution and is no longer bonded to the Cu^2+^ ion.^[^ ^39^ ^]^ Therefore, it can be concluded that the Cu^2+^ ion dissociates from Cu 2‐EHA in solution and forms a metal‐complex bond with the α‐carbon of thiophene through the π electrons of the C═C bond, which is visually identifiable by the pale yellow color of the solution (Figure 1b). According to condensed‐phase studies, thiophene exhibits both phi‐bonding mode (C═C) and sigma‐bonding (C─S) mode. Molecular orbital calculations conducted by Sargent et al.^[^ ^40^ ^]^ indicate that the coordination strength of the ligand depends on the magnitude and polarizability of the electronic density of sulfur's lone pair electrons. The delocalization of the lone pair electron density achieved through conjugation with adjacent phi bonds decreases the Lewis basicity of sulfur and makes thiophene a poor sigma‐bonding ligand. Consequently, thiophene prefers interactions with metals through phi conjugate bonds (C═C) rather than sulfur lone pair electrons. In contrast, in the case of tetrahydrothiophene (THT), the metal complex formation with THT occurs only through lone pair electrons of sulfur.^[^ ^18^ ^]^ As the π bond is absent in THT, no delocalization of lone pairs happens, as a result, sulfur in THT has high electron density which results in at least 7.1 × 10^6^ times stronger metal complexes in THT compared to thiophene.^[^ ^41^ ^]^ The delocalized π electrons of the C═C bond in thiophene act as a ligand, causing splitting of the d‐orbital of the Cu^2+^ ion and an electronic energy transition occurs from lower energy T_2g_ to higher energy E_g_ level.^[^ ^42^ ^]^ This leads to an increased absorption peak from the wavelength 580 nm (Figure 1b), which is related to the d‐d transition in the orbitals of the metal Cu^2+^.^[^ ^43^ ^]^ The small shoulder peak at (350–400) nm (Figure 1b) is associated with the π–π* transition from the C═C bond.^[^ ^44^ ^]^

Figure 1c illustrates the difference in spectra between THF and the mixture of THF and Cu 2‐EHA. If these difference spectra predominantly originate from Cu 2‐EHA, it suggests that the interaction between Cu 2‐EHA and THF is absent but if the difference spectra mainly arise from THF, it indicates that there is an interaction between Cu 2‐EHA and THF. The difference spectra show sharp peaks at 1615 and 1419 cm^−1^, corresponding to the Cu 2‐EHA derived asymmetric and symmetric vibration of COO^−^ respectively. Some small difference spectra appear at ≈1325 cm^−1^ related to the CH_3_ deformation peak and at ≈(821–652) cm^−1^ range, associated with the ─CH₂ twist vibration of Cu 2‐EHA. As the difference peaks originate only from Cu 2‐EHA, it is concluded that the coordination bond between the Cu^2+^ ion and THF solution is absent. There is a broad peak at ≈(600–700) nm in the UV–vis absorption peak of Cu 2‐EHA and THF solution which is related to the color of the Cu 2‐EHA (Figure 1d). There is no characteristic peak observed, which is attributed to the interaction between Cu 2‐EHA and THF. Therefore, the UV–vis absorption result of the Cu 2‐EHA and THF solution reinforces the conclusion of the absence of interaction between the Cu^2+^ ion and THF. These phenomena can be explained by the hard and soft acid and base (HSAB) principle, proposed by Pearson.^[^ ^45^ ^]^ This principle states that hard acids, primarily metallic ions, exhibit a preference for interacting with hard bases, which are typically donor atoms while soft acids have the propensity to interact with soft bases. Hard acids and hard bases, characterized by low polarizability, tend to engage in electrostatic interactions, while soft acids and bases, with higher polarizability, exhibit interactions of a more covalent nature. According to this principle, Cu^2+^ is a borderline acid that has almost no interaction with a hard base like THF.^[^ ^46^ ^]^

The FTIR analysis of the mixture of thiophene and Cu 2‐EHA (Figure 1a) shows the C─S spectrum at 833 cm^−1^, identical to that found in the pure thiophene solution spectrum. This implies that the C─S bond is intact in the precursor solution and interaction between the Cu and sulfur is absent in the solution. Hence, sulfur release and copper sulfide formation exclusively occur in the gas phase after spraying the solution.^[^ ^47^ ^]^

### 2.2 Effect of Fuel‐To‐Oxygen Ratio (*ϕ*) on the Copper Sulfide Synthesis Process

**Figure 2:**
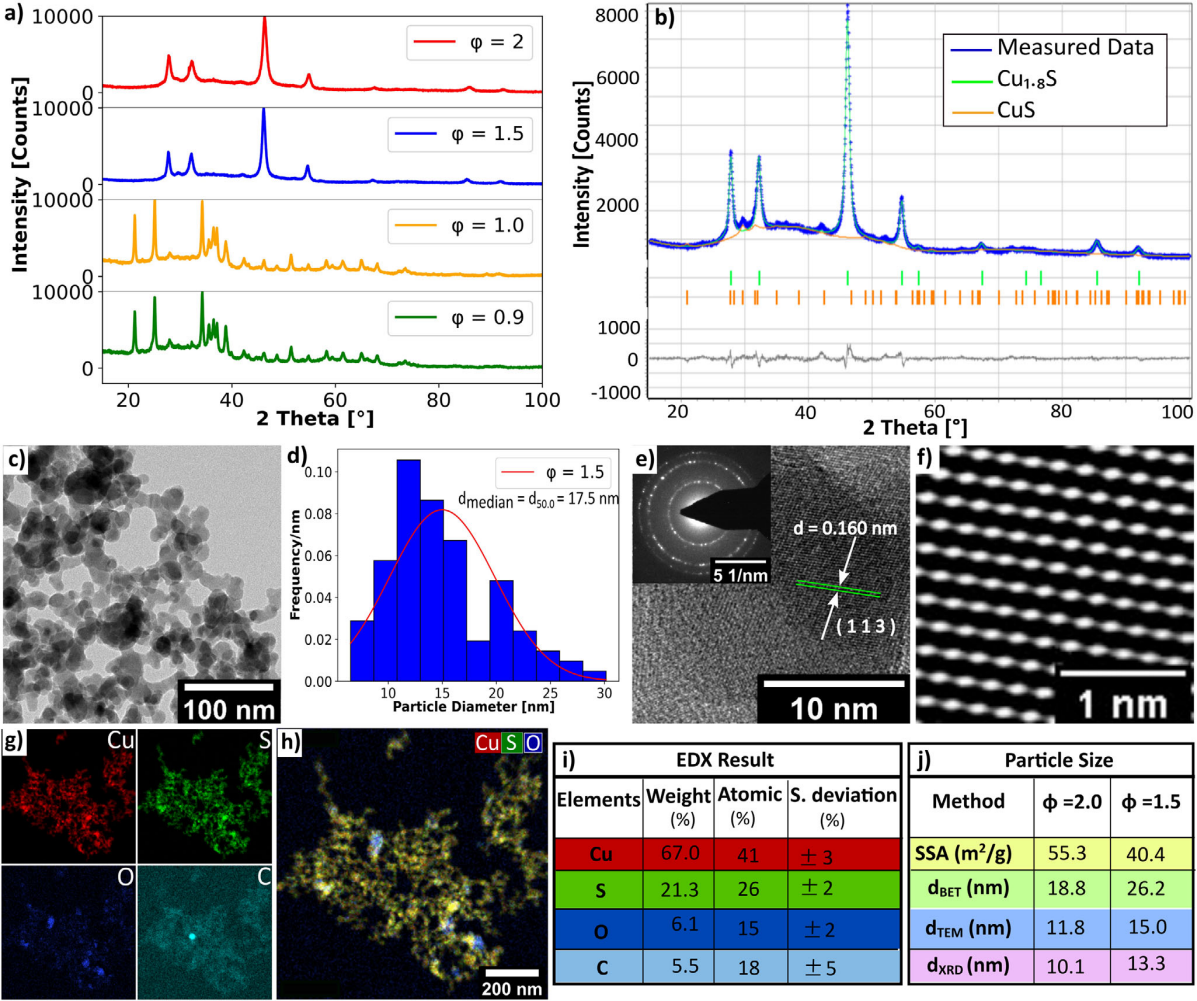
a) XRD reflections of copper sulfide prepared with different *ϕ* values are shown. Distinctive reflections for Cu_1.8_S are observed at 27.9°, 32.3°, 46.4°, 54.9°, and 86.1° for *ϕ* = 2.0 and 1.5 but for *ϕ* = 1.0 and 0.9, Cu_1.8_S reflections are invisible. b) Rietveld refinement of the *ϕ* = 1.5 sample. The refinement was performed with both cubic Cu_1.8_S (ICSD 41 142, F m ‐3 m, a = 5.539 Å) and hexagonal CuS (ICSD 41 911, P 63/m m c, a = 3.8220 Å, c = 16.115 Å) phases using BRASS software. Refinement parameters are *R_exp_* = 3.14, *R_p_* = 3.65, and *R_wp_* = 4.52. c) The bright‐field image shows an aggregate of approximately spherically shaped Cu_1.8_S particles. d) Particle size distribution of the *ϕ* = 1.5 sample is shown, which was calculated from a low‐resolution TEM image. e) Atomic resolution TEM image and Debye‐Scherrer diffraction pattern of Cu_1.8_S indicate the polycrystalline nature of the particle. f) Fourier‐filtered HRTEM image. g) Color maps of the net EDX intensity of Cu, S, O, and C. h) Color mix maps of the net intensity of Cu, S, and O of an aggregate. Due to the selection of the colors, a yellow color indicates Cu_2_S particles, whereas blueish particles are oxygen elements. i) Elemental concentration determination from STEM‐EDX shows that particle composition matches with Cu_1.8_S approximately. j) With decreasing *ϕ* value from 2.0 to 1.5, the specific surface area (SSA) decreases, while the particle diameter *d_BET_*, *d_TEM_*, and crystallite size *d_XRD_* increase.

Maintaining an accurate fuel‐rich condition is a crucial requirement for the metal sulfide formation in the reducing flame. The degree of oxidization can be regulated by the fuel‐to‐oxygen ratio (*ϕ*), which is defined as the ratio of the moles of oxygen required for the complete combustion of the metal precursor and solvents to the moles of oxygen supplied in the flame. As the flame is enclosed by a quartz tube, entrainment of air from the environment is impossible. Thus, the amount of oxygen in the flame can be controlled by the supplied dispersion oxygen flow. To investigate the effect of fuel‐to‐oxygen ratio on copper sulfide formation, the precursor solution was atomized using varying dispersion oxygen flows corresponding to *ϕ* = 2.0, 1.5, 1.0, and 0.9 (according to the definition as *ϕ* decreases the dispersion oxygen flow increases), while maintaining a constant feed rate of 5 mL min^−1^, a co‐flow of 200 L min^−1^, and a Cu to S molar ratio of 1:30 in the precursor solution. **Figure** **2**a shows Cu_1.8_S reflections for *ϕ* = 2.0 and *ϕ* = 1.5 but for *ϕ* = 1.0 and 0.9 reflections for Cu_1.8_S is invisible. In the case of *ϕ* = 1.0 and *ϕ* = 0.9, the dispersion oxygen flows are 8.3 and 9.22 L min^−1^, respectively which are higher than those for *ϕ* = 2.0 and 1.5. As more oxygen is supplied to the flame, a greater quantity of the precursor is available for complete combustion, resulting in higher temperatures within the reaction zone. The temperature of the aerosol stream exiting the glass tube is 420 and 450 °C for *ϕ* = 1.0 and 0.9 respectively, and at this temperature, Cu_1.8_S is converted mainly into copper sulfate hydroxide hydrate.^[^ ^48^ ^]^ Therefore, when the fuel‐to‐oxygen ratio is equal to or lower than 1, Cu_1.8_S may initially form as an intermediate product but it transforms immediately to copper sulfate hydroxide hydrate due to the high temperature. Rietveld refinement of the as‐prepared copper sulfide with *ϕ* = 1.5 revealed 95.85 wt.‐% Cu_1.8_S and 4.15 wt.−% CuS phases (Figure 2b).

The bright‐field TEM image of Figure 2c shows an aggregate of almost spherically shaped Cu_1.8_S nanoparticles. Figure 2d shows a Gaussian particle size distribution of the sample with *ϕ* = 1.5, indicating a median particle diameter of 17.5±4.9 nm. The atomic resolution TEM image of Cu_1.8_S and Debye–Scherrer diffraction pattern (Figure 2e) indicate the polycrystalline nature of the particles. The distance between the two crystal planes is 0.160 nm which signifies the (1 1 3) plane orientation. Figure 2f shows a Fourier‐filtered high‐resolution transmission electron microscopy (HRTEM) image of the nanoparticle.

Figure 2g–i show colormap, mixed colormap, and EDX results of the quantitative elements of Cu, C, O, and S in the sample. As the dispersion oxygen increases from 4.15 to 5.53 L min^−1^, the oxygen content in the nanoparticle also increases from 2.82 to 3.21 wt.‐%. So, to minimize the oxygen impurity in the sample, *ϕ* = 2.0 (dispersion oxygen 4.15 L min^−1^) is recommended because it provides enough dispersion oxygen gas for atomization while minimizing the oxygen impurity. Figure 2j shows an increase of the *d_BET_*, *d_TEM_*, and *d_XRD_* with decreasing phi value (increasing dispersion oxygen). Simultaneously, the temperature of the exiting aerosol stream increases from 300 to 350 °C as the *ϕ* value decreases from 2.0 to 1.5. As the temperature increases, the particle resides in the higher‐temperature flame, which results in a higher sintering rate. Thus, the primary diameter of the particle increases as the *ϕ* value decreases. This result is inconsistent with the metal oxide production in FSP.^[^ ^1b^ ^]^

In a typical setup of FSP for metal oxide production, increasing the dispersion gas flow rate intensifies the mixing, which reduces both the droplet diameter and the concentration of particles in the flame.^[^ ^1b^ ^]^ This also leads to wider and shorter flames.^[^ ^49^ ^]^ Thus, particle concentration and residence time at high‐temperature decrease as the spray flame height decreases, with increasing dispersion gas flow rate. This leads to faster quenching of particle formation and therefore smaller particles. However, in the case of metal sulfide production under reducing flame conditions, even though the dispersion gas increases the mixing of the precursor, it can not accelerate the combustion rate because the supplied oxygen is far less than what is required for complete combustion. So, when more dispersion gas is supplied, it enables more precursor to go for complete combustion, as a result, it increases both the temperature and height of the flame. Hence, here the supply of dispersion oxygen is the controlling factor for combustion. If the dispersion gas increases, more oxygen becomes available for combustion, which increases the total heat produced due to combustion and flame temperature significantly. As the particle resides a longer time in the high‐temperature flame, particle diameter increases with increasing dispersion of oxygen flow.

### 2.3 Effect of Cu to S Molar Ratio on the Copper Sulfide Synthesis Process

**Figure 3:**
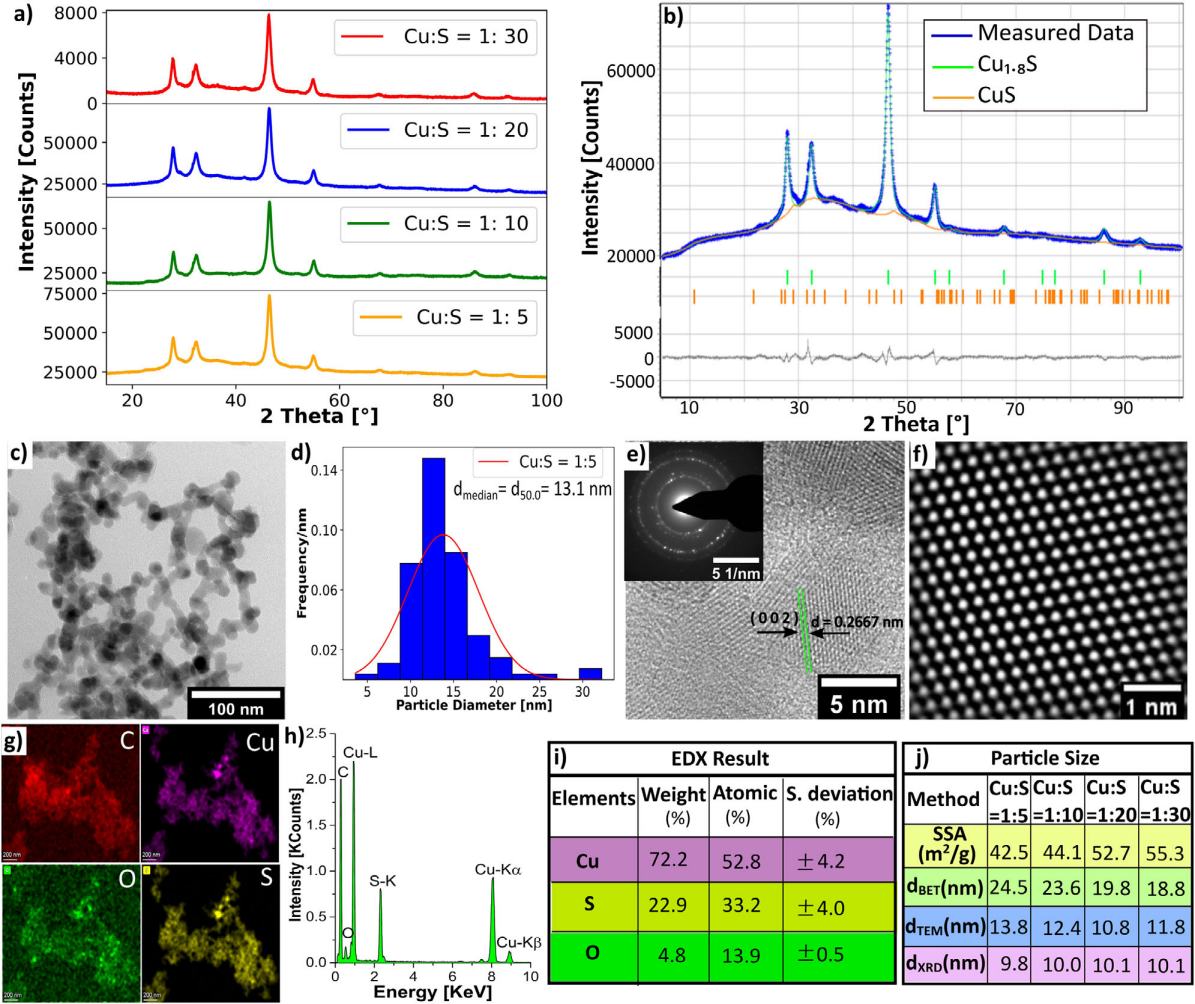
a) X‐ray diffraction reflections of copper sulfide particles prepared with Cu to S molar ratios of 1:5, 1:10, 1:20, and 1:30 in the precursor solution, showing distinguishable Cu_1.8_S reflections for all samples. b) Rietveld refinement of the Cu:S = 1:5 sample reveals a composition of Cu_1.8_S and CuS phases respectively 97.04 and 2.96 wt.‐% with refinement results of *R_exp_* = 0.62, *R_p_* = 0.84, and *R_wp_* = 1.08. c) The bright‐field image of TEM shows aggregates of approximately spherically shaped Cu_1.8_S nanoparticles. d) The particle size distribution of Cu:S = 1:5 sample is shown, with a median diameter of 13.1±3.6 nm. e) Atomic resolution TEM image of Cu_1.8_S particle and Debye–Scherrer diffraction pattern indicates the polycrystalline nature of the particles. The distance between the two crystal planes is 0.2667 nm which represents the (0 0 2) plane orientation. f) Fourier‐filtered HRTEM image. g) Color maps of the net EDX intensity of Cu, S, O, and C. h) The intensity distribution of the elements is shown with their respective reflections. i) Elemental concentration determination from STEM‐EDX shows that particle composition matches with Cu_1.8_S approximately. j) Specific surface area (SSA) increases and *d_BET_* and *d_TEM_* decrease with increasing Cu to S molar ratio, while the crystallite sizes, *d_XRD_* calculated from XRD slightly increase.

For metal sulfide formation, a minimum ratio of metal‐to‐sulfur is necessary because naturally metals have a higher affinity for oxygen than for sulfur. At high temperatures, thiophene decomposes to mainly H_2_S.^[^ ^50^ ^]^ At higher temperatures, H_2_S is absorbed by the metal oxide intermediate compound,^[^ ^51^ ^]^ and produces metal‐sulfide components. To ensure an adequate supply of H_2_S in the vicinity of metal‐oxide at higher temperatures, a certain minimum sulfur concentration is required in the precursor solution. To investigate the influence of the Cu to S molar ratio in the copper sulfide synthesis process, precursor solutions with different Cu to S molar ratios, specifically 1:5, 1:10, 1:20, and 1:30 were prepared and sprayed at a constant feed rate of 5 mL min^−1^, a fuel‐to‐oxygen ratio of *ϕ* = 2, and a co‐flow rate of 200 L min^−1^. It is experimentally found that for the copper sulfide formation, a minimum Cu to S ratio of 1:5 is sufficient. **Figure** **3**a shows the XRD reflections, providing evidence of Cu₁.₈S particle formation at different Cu to S ratios. The Rietveld refinement of the Cu:S = 1:5 sample (Figure 3b) reveals a composition of 97.04 wt.‐% Cu_1.8_S and 2.96 wt.‐% CuS phases. The results show the absence of correlation between the phase composition of the nanoparticle and Cu to S ratio in the precursor solution. The bright‐field image of TEM (Figure 3c) shows aggregates of approximately spherically shaped copper sulfide nanoparticles. Particle size distribution of the Cu:S = 1:5 sample gives a median diameter of 13.1±3.6 nm (Figure 3d). Atomic resolution TEM image of Cu_1.8_S particles and Debye–Scherrer diffraction pattern (Figure 3e) indicate the polycrystalline nature of the particles. The distance between the two crystal planes is 0.2667 nm which corresponds to the (0 0 2) plane orientation. Fourier‐filtered HRTEM image (Figure 3f) reveals the periodic arrangement of atomic layers and allows clear identification of the (0 0 2) planes. Figure 3g–i show respectively the colormap, intensity, and quantitative results of the EDX analysis of the elements for the Cu:S = 1:5 sample as a representative for different Cu:S ratio samples.

As all the samples were synthesized with the same dispersion oxygen flow rate, the content of oxygen in all the samples is almost identical across the different Cu:S ratio samples. As the Cu:S ratio increases from 1:5 to 1:30, the sulfur content (wt.%) also increases from 33.25% to 37.17%. This increase in the sulfur element could introduce pure sulfur particles that are not bonded to Cu in the sample. Figure 3j shows a decrease in *d_BET_* from 24.5 nm to 18.8 nm and in *d_TEM_* from 13.8 nm to 11.8 nm with increasing Cu to S molar ratio from 1:5 to 1:30. Specific surface area increases from 42.52 to 55.3 m^2^ g^−1^ with the increasing Cu to S molar ratio from 1:5 to 1:30. As the concentration of the sulfur increases in the reaction zone, H_2_S release increases and it augments the supersaturation level of H_2_S in the gas phase.^[^ ^52^ ^]^ Increasing supersaturation levels mean an increasing number of nucleation sites, which favors the formation of a large number of nuclei.^[^ ^53^ ^]^ For a given concentration of a solute, a large number of nuclei means small‐sized nuclei. Therefore, increasing sulfur concentration leads to smaller nuclei and consequently smaller primary particle diameter. Moreover, as the Cu:S ratio increases from 1:5 to 1:30, the thermal energy supplied to the flame increases from −31.99 to −34.20 KJ mL^−1^, contributing to an increase in the exiting aerosol temperature from 280 to 300 °C. This temperature increase is too low to influence particle growth. However, the slight temperature rise is one of the reasons for the modest increase in the crystallite size, *d_XRD_* from 9.8 nm to 10.1 nm. As the temperature increases, adjacent nanoparticles fuse to form larger crystallites.^[^ ^54^ ^]^

### 2.4 Effect of Precursor Feed Rate on the Copper Sulfide Synthesis Process

**Figure 4:**
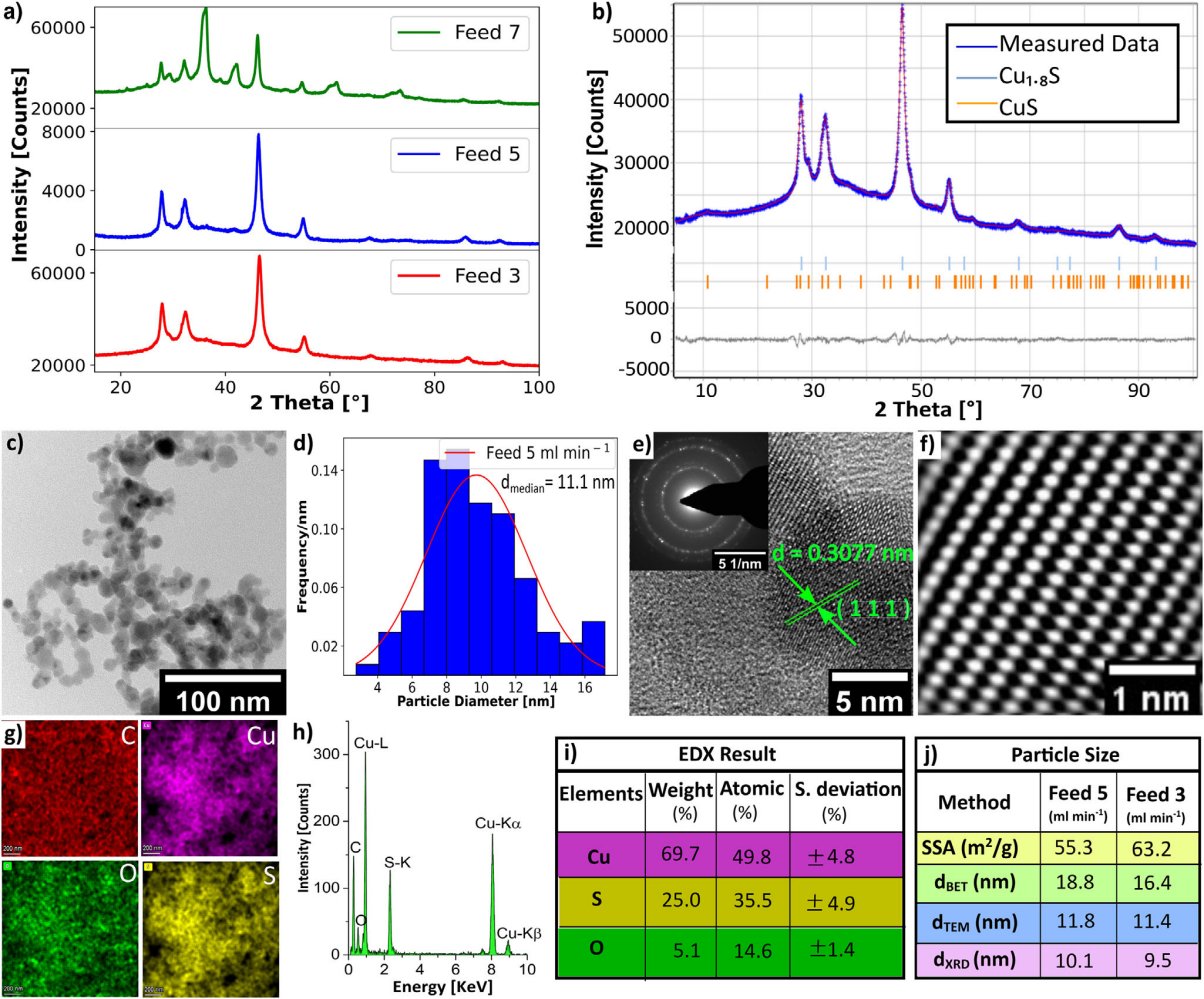
a) XRD reflections of copper sulfide prepared with 3, 5, and 7 mL min^−1^ precursor feed rates. The distinctive reflections for Cu_1.8_S are observed at feed rates of 5, and 3 mL min^−1^ but for 7 mL min^−1^, the reflections for Cu_1.8_S are invisible. b) Rietveld refinement of the feed 5 mL min^−1^ sample as a representative for different feed rate samples. The refinement was performed for both cubic Cu_1.8_S and hexagonal CuS phases, which reveals a composition of 97.45 wt.‐% Cu₁.₈S and 2.55 wt.‐% CuS, with refinement results of *R_exp_* = 0.62, *R_p_* = 0.86, and *R_wp_* = 1.16. c) The bright‐field image shows an aggregate consisting of approximately spherically shaped Cu_1.8_S. d) Particle size distribution of the feed rate 5 mL min^−1^ sample, with a median diameter of 11.1 nm, calculated from the TEM image. e) Atomic resolution TEM image of Cu_1.8_S particle and Debye‐Scherrer diffraction pattern indicates the polycrystalline nature of the particle. The distance between the two crystal planes is d = 0.307 nm which indicates the (1 1 1) plane orientation. f) Fourier‐filtered HRTEM image. g) Color maps of the net EDX intensity of Cu, S, O, and C. h) The Intensity distribution of the elements with their respective reflections. i) Elemental concentration determination from STEM‐EDX shows that particle composition matches with Cu_1.8_S approximately. j) Specific surface area (SSA) increases, while particle size *d_BET_*, *d_TEM_*, and crystallite size *d_xrd_* are almost the same with decreasing feed flow rate from 5 to 3 mL min^−1^.

To investigate the influence of precursor feed rate on the copper sulfide synthesis process, the precursor solution was introduced at rates of 3, 5, and 7 mL min^−1^ while maintaining a constant fuel‐to‐oxygen ratio *ϕ* = 2.0, a co‐flow rate of 200 L min^−1^, and a Cu to S molar ratio of 1:30 in the precursor solution. **Figure** **4**a shows XRD reflections of Cu_1.8_S for precursor feed rates 3 and 5 mL min^−1^ but in the case of 7 mL min^−1^, the XRD reflections corresponding to the Cu_1.8_S phase are unnoticeable. During 7 mL min^−1^ feed rate, the supplied enthalpy to the flame reaches −239.42 KJ min^−1^ which leads to an aerosol stream temperature of ≈520 °C. At this temperature, Cu_1.8_S becomes unstable and converts into copper sulfide hydroxide hydrate.^[^ ^48^ ^]^

Figure 4b shows the Rietveld refinement of the 5 mL min^−1^ feed rate as a representative for different feed rate samples. The refinement was performed using both cubic Cu_1.8_S and hexagonal CuS phases, revealing a composition of 97.45 wt.‐% Cu₁.₈S and 2.55 wt.‐% CuS. The bright‐field TEM image (Figure 4c) shows an aggregate consisting of approximately spherically shaped Cu_1.8_S nanoparticles. Figure 4d shows the particle size distribution of 5 mL min^−1^ feed rate sample, with a median diameter of 11.1 ± 3.78 nm, calculated from the TEM image. The Atomic resolution TEM image of Cu_1.8_S particle and Debye–Scherrer diffraction pattern (Figure 4e) indicate the polycrystalline nature of the particle. The distance between two crystal planes is d = 0.307 nm, corresponding to the (1 1 1) plane orientation. The Fourier‐filtered HRTEM image (Figure 4f) highlights the crystalline structure of the phase pure nanoparticle. Figure 4g–i shows the colormap, intensity distribution, and EDX results for Cu, S, and O elements. As the precursor feed rate increases from 3 to 5 mL min^−1^, the oxygen content in the particles increases from 4.66 to 8.97 wt.‐%. Increasing the feed rate from 3 to 5 mL min^−1^ also increases the exiting aerosol temperature from 210 to 300 °C. As the temperature increases with increasing feed rate, copper sulfide particles become more reactive, and adsorbed oxygen diffuses in the sulfide lattice, which increases the oxygen content in the particle.^[^ ^55^ ^]^ Even though a higher feed rate leads to a higher production rate,^[^ ^56^ ^]^ it introduces more impurities into the copper sulfide particle, and beyond a certain limit of 7 mL min^−1^, the copper sulfide phase undergoes a complete transformation. Figure 4j shows that the change of the *d_BET_*, *d_TEM_*, and *d_XRD_* with increasing feed rate from 3 mL min^−1^ to 5 mL min^−1^ is insignificant. Hence, increasing the feed rate doesn't influence the particle diameter.

### 2.5 Effect of Co‐Flow (N_2_ flow) on the Copper Sulfide Synthesis Process

**Figure 5:**
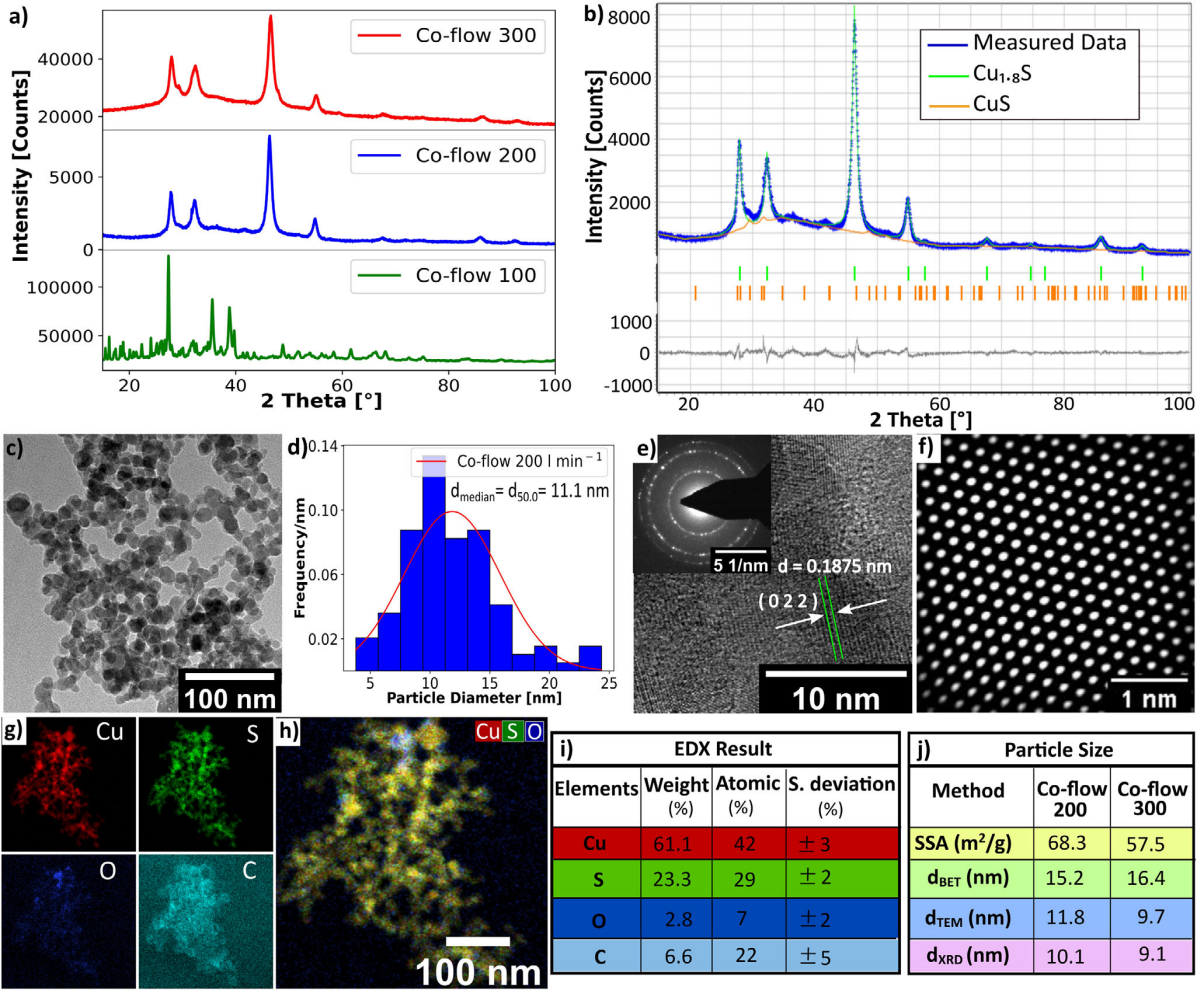
a) XRD reflections of copper sulfide prepared with 100, 200, and 300 L min^−1^co‐flow of N_2_. Distinctive reflections for Cu_1.8_S are observed at co‐flow 200 and 300 L min^−1^ but for co‐flow 100 L min^−1^ the reflections for Cu_1.8_S are invisible. b) Rietveld refinement of co‐flow 200 L min^−1^ sample is shown. The refinement was performed using both cubic Cu_1.8_S and hexagonal CuS phases, yielding a composition of 92.23 wt.‐% Cu₁.₈S and 7.77 wt.‐% CuS, with refinement results of *R_exp_* = 0.66, *R_p_* = 0.76, and *R_wp_* = 0.98 c) The bright‐field image shows an aggregate consisting of approximately spherically shaped Cu_1.8_S. d) Particle size distribution of the co‐flow 200 L min^−1^ sample, showing a median diameter of 11.1± 3.79 nm, calculated from the low‐resolution TEM image. e) Atomic resolution image of Cu_1.8_S particle and Debye–Scherrer diffraction pattern indicate the polycrystalline nature of the particle. The distance between the two crystal planes is d = 0.187 nm, corresponding to the (0 2 2) plane orientation. f) Fourier‐filtered HRTEM image. g) Color maps of the net EDX intensity of Cu, S, O, and C. h) Color mixed maps of the net intensity of Cu, S, and O of an aggregate. i) Elemental concentration determination from STEM‐EDX shows that particle composition matches with Cu_1.8_S approximately. j) Specific surface area (SSA) increases, while particle size *d_BET_*, *d_TEM_*, and crystallite size *d_xrd_* decrease with increasing co‐flow rate.

To investigate the effect of N_2_ co‐flow rates on the copper sulfide synthesis process, co‐flow rates of 100, 200, and 300 L min^−1^ were introduced while maintaining a constant feed rate of 5 mL min^−1^, a fuel‐to‐oxygen ratio of *ϕ* = 2.0, and ensuring a Cu to S molar ratio of 1:30 in all precursor solutions. **Figure** **5**a shows the X‐ray diffraction reflections of Cu_1.8_S for co‐flow rates of 200 and 300 L min^−1^ but for 100 L min^−1^, the pure copper sulfide is converted to different phases because at 100 L min^−1^ co‐flow rate, the exiting aerosol stream temperature reached 630 °C, at which Cu_1.8_S become unstable and converts into copper sulfide hydroxide hydrate.^[^ ^48^ ^]^ Figure 5b shows the Rietveld refinement of the co‐flow 200 L min^−1^ sample and the refinement results show no correlation with co‐flow and phase composition of the nanoparticles. Figure 5c represents a bright‐field TEM image of a Cu_1.8_S aggregate, which consists of approximately spherically shaped nanoparticles. The particle size distribution of the 200 L min⁻¹ co‐flow sample (Figure 5d) reveals a median diameter of 11.1 ± 3.79 nm, calculated from the low‐resolution TEM image. The atomic‐resolution TEM image and the Debye–Scherrer diffraction pattern of the Cu_1.8_S particle (Figure 5e) confirm the polycrystalline nature of the particle. The distance between the two crystal planes is d = 0.187 nm, corresponding to the (0 2 2) plane orientation. The fourier‐filtered HRTEM image (Figure 5f) enhances the visualization of lattice fringes, revealing the periodic arrangement of atomic layers within the nanoparticle. Figure 5g–i shows the colormap, intensity distribution, and EDX results of the Cu, S, and O elements for co‐flow 200 L min^−1^ as a representative for different co‐flow samples. The EDX results reveal an absence of any correlation between the elemental composition and co‐flow rate due to maintaining constant dispersion oxygen supply, feed rate, and Cu to S ratio in the solution for different co‐flow rates. Figure 5j shows a decrease in *d_BET_*, *d_TEM_*, and crystallite size *d_xrd_* with an increasing co‐flow rate from 200 to 300 L min^−1^ which corresponds to decreasing aerosol temperature from 300 to 214 °C. Increasing the co‐flow rate decreases the exiting aerosol temperature which reduces the nucleation and growth rates of the nanoparticle and lower growth rates can lead to smaller primary particle diameter.^[^ ^19^ ^]^

Moreover, increasing the co‐flow rate accelerates the convective heat transfer between the flame and the surroundings,^[^ ^57^ ^]^ which reduces strong vortex formation and recirculation zone which leads to a decrease in both the particle concentration and particle residence time inside the high‐temperature flame. As a result, a smaller primary nanoparticle diameter is obtained at higher co‐flow.

## 3 Potential Application of FSP‐Synthesized Copper Sulfide

**Figure 6:**
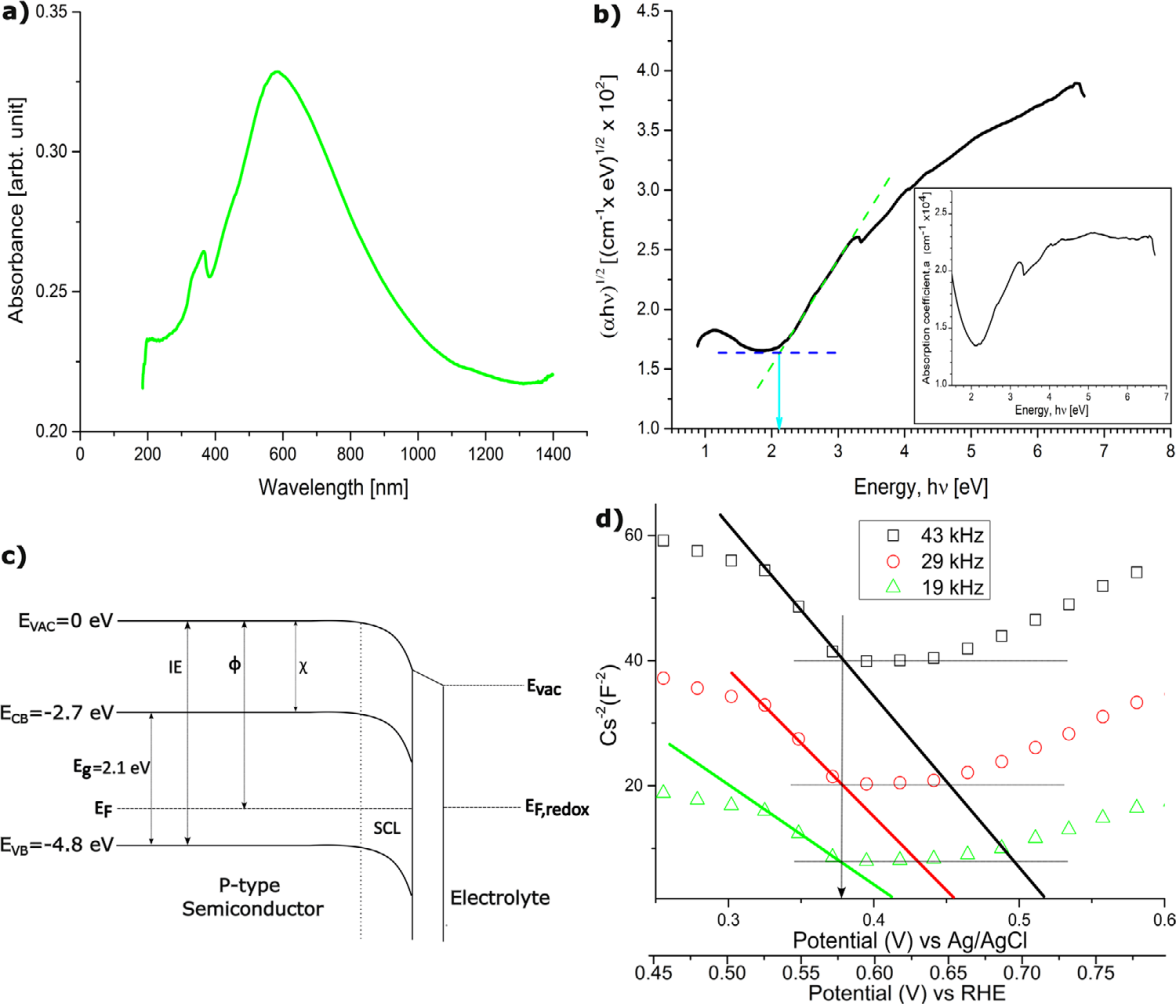
a) UV–vis absorption spectrum of the as‐prepared Cu_1.8_S nanoparticle (*ϕ* = 2.0, precursor flow rate: 5 mL min^−1^, co‐flow rate: 200 L min^−1^), which shows broad absorption peak at the visible and NIR region. b) The direct optical bandgap was determined from the Tauc plot using the Kubelka–Munk function. The inset plot is the absorption coefficient versus the photon energy of the as‐prepared nanoparticle. C) The position of the valence band, E_VB_ (−4.8 eV), and conduction band, E_CB_ (−2.7 eV) edges are shown with respect to the absolute vacuum scale. A bandgap is determined as 2.1 eV. D) Mott−Schottky plots obtained at 19, 29, and 43 kHz, which show a flat band potential of 0.38V versus Ag/Agcl (pH 6.7) or 0.58V versus RHE.

UV–vis absorption spectroscopy is the most utilized technique to determine the optical characteristics of semiconductor nanomaterials. **Figure** **6**a shows a UV–vis absorption spectrum of the as‐prepared copper sulfide nanoparticle. The figure shows a broad spectrum in the spectral region between 400 to 800 nm. The increase of absorption peak at the beginning of 400 nm is associated with the transition from the valence band to the conduction band. There is also a wide shoulder at ≈610 nm in the near‐infrared region (NIR) region, due to high free‐carrier absorption derived from holes in the valence band.^[^ ^58^ ^]^ As the data clearly shows a broad absorption spectrum in both visible and NIR regions, our synthesized copper sulfide is a promising material for solar cells and photo‐catalysis applications.^[^ ^59^ ^]^ The bandgap energy (E_g_) of a semiconductor material fundamentally determines the light absorption ability and subsequently influences the solar energy conversion efficiency. So, determining the bandgap accurately is one of the crucial steps for the design and development of photo‐catalysts.

The efficiency of a semiconductor cell is maximized if the incident photon energy matches or exceeds the bandgap energy of the material. When the material absorbs photons with wavelengths corresponding to the energy gap between its valence and conduction bands, there is a sudden rise in absorbance. The energy corresponding to the wavelength where this absorption starts to rise indicates the minimum energy needed for a photon to excite an electron across the bandgap, allowing it to be absorbed by the semiconductor material. The nonlinear increase in absorption observed in real spectra (Figure 6b) suggests variation in the local density of states at the conduction band minimum and valence band maximum, along with the presence of excitonic effects.^[^ ^60^ ^]^ The commonly used expression to determine the bandgap from UV–vis absorption edge of semiconductors is based on the following Tauc relation

(1) $$\varepsilon h\upsilon =A\;{\left(h\upsilon \;-\;\mathrm{Eg}\right)}^{n}$$

where ɛ is the molar extinction coefficient, A is a constant, E_g_ is the optical bandgap of the sample and hʋ is the incident photon energy.

The value of *n* varies based on the type of transition, n = ½ for direct allowed transitions and n = 2 for indirect allowed transitions. The bandgap value calculated from the Tauc plot (Figure 6b) by considering n = ½ is 2.1 eV. This bandgap energy is equal to the energy of the onset of the rising absorption coefficient of the material which indicates that n = ½ is the right choice and copper sulfide constitutes a direct bandgap. Copper sulfide prepared in other literature with a bandgap of 2.31 eV demonstrates high sensitivity to ammonia gas and a short response time of 15s at both room temperature and higher temperatures.^[^ ^61^ ^]^ As the sensitivity of the gas sensor is primarily a function of the bandgap of the material,^[^ ^62^ ^]^ the FSP‐synthesized copper sulfide in this study with a lower bandgap of 2.1 eV has potential applications as an ammonia gas sensor. Moreover, due to its narrow bandgap and good optical absorption efficiency in the visible to NIR region, copper sulfide is a promising material for visible light‐responsive photocatalysts with enhanced solar energy conversion.^[^ ^23b^ ^]^

Understanding the relationship between semiconductor and electrolyte energy levels is one of the most important factors for the development of electrochemical photovoltaic cells. The electrocatalytic ability of a semiconductor depends on its electron injection ability of the material at the surface, which is determined by the energetic characteristics of the conduction and valence bands. Hence, determining the absolute position of the E_CB_ and E_VB_ band edges is necessary to explore the potential applications of the semiconductor material. One of the important parameters to determine the electrochemical potential of electrons in a semiconductor is the fermi level E_F_, which is defined as the thermodynamic equilibrium energy level that has a 50% probability of being occupied by an electron at any given time. The position of the fermi level in relation to the valence and conduction band determines the nature of charge carrier mobility. The energy of a stationary electron located in close proximity to the surface of the semiconductor relates to the vacuum level (E_vac_), which is the pivotal reference point that defines all other parameters as illustrated in Figure 6c. The energy needed to excite an electron from E_VB_, E_CB_, E_F_ to E_VAC_ is known as ionization energy, electron affinity, and work function respectively.

When a semiconductor is in contact with an electrolyte, the ionic interaction in the semiconductor‐electrolyte interface depends on the energetic position of the semiconductor E_f_ and electrolyte E_f, redox_. Electron flows from the more negative phase of E_f_ to the other side to achieve equilibrium until the semiconductor E_F_ matches with the electrolyte E_f, redox._ This causes the formation of the space charge layer (SCL) within the semiconductor material which is attributed to the downward band bending of the p‐type semiconductor material. The magnitude and direction of the band bending can be simply adjusted by an externally applied potential. The applied bias potential that diminishes the band bending in a semiconductor that is in contact with the electrolyte is called flat band potential (U_FB_). One of the most common experimental techniques to determine the valence and conduction band edge position based on flat band potential measurement is via electrochemical impedance spectroscopy (EIS) methods using the Mott–Schottky function. It also offers the quantitative determination of charge transport and trapping in porous nanoparticle electrodes.

To determine the flat band potential U_FB_ of copper sulfide nanoparticles, EIS analysis was performed for the nanoparticle‐polymer electrodes. According to the Mott–Schottky relation, the space‐charge capacitance C varies as a function of the applied voltage U:

(2) $$\frac{1}{C^{2}}=\frac{2}{\varepsilon_{r}\varepsilon_{0}eA^{2}N_{D}}\left(U-U_{\mathrm{FB}}-\frac{K_{B}T}{e}\right)$$

where N_D_ is the majority charge carrier density, e is the electron charge, and ɛ_r_ and ɛ_0_ are the dielectric constant of the material and the vacuum permittivity respectively. A is the surface area of the electrode, K_B_ is the Boltzmann constant, and T is the absolute temperature. The intersection point of a Mott–Schottky plot (1/C^2^ vs U) with the x‐axis gives the flat band potential U_FB_. The slope of the plot contains information on the charge carrier density N_D_ and allows calculating the difference between the valence band and the Fermi level in p‐type semiconductors. For the Mott–Schottky analysis, 19, 29, and 43 kHz frequencies were chosen so that high‐frequency measurement has the most effect on the flat band potential measurement. The plot in Figure 6d shows the same intersection point with the baseline independent of the frequency chosen, which indicates a reproducible flat band potential of 0.38V versus Ag/Agcl (pH 6.7) or 0.58V versus RHE.

Electronegativity decreases from 3.44 to 2.58 when moving down group VI of the periodic table from O to S. As a result, the Cu─S bond in the Cu_2_S is less ionic compared to the Cu─O bond in Cu_2_O. Since Cu has high‐energy 3d orbitals, it promotes a larger overlap of the d‐p orbital in Cu_2_S compared to Cu_2_O. Thus, 3p orbitals form S hybridize more strongly with Cu 3d orbital.^[^ ^63^ ^]^ Due to the more delocalized nature of the S 3p orbital compared to the O 2p orbital, hole carriers of the metal sulfide have less effective mass than those in the metal oxide which enables higher charge carrier mobility of metal sulfide than metal oxide.^[^ ^64^ ^]^ In general, in the case of copper sulfide, the Cu 3d and S 3p orbitals contribute to the valence band edge maximum, which is determined as −4.8 eV versus AVS (Absolute Vacuum Scale).^[^ ^63^ ^]^ The Conduction band edges originate mainly from Cu 3d orbitals, the calculated bandgap (2.1 eV), and the valence band position. The conduction band edges are estimated as −2.7eV versus AVS. Moreover, the delocalization nature of the metal sulfide orbital leads to a narrower bandgap compared to metal oxide. As a result, metal sulfide can absorb both visible and UV light, whereas metal oxide can only use UV light which is only 3% of the solar radiation.

## 4 Conclusion

**Figure 7:**
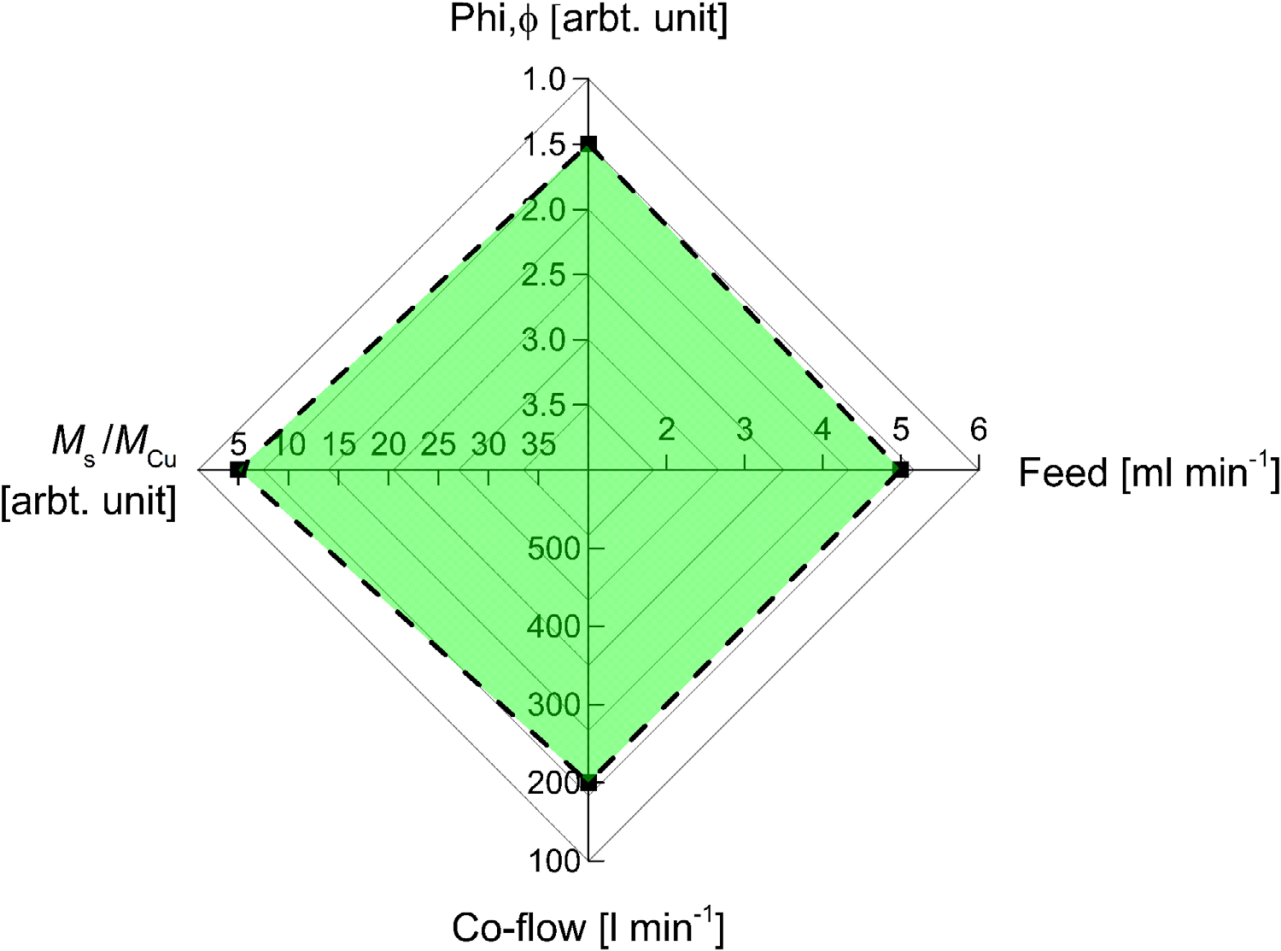
This figure summarizes the boundary of the operating parameters for copper sulfide production in an enclosed flame FSP setup. Any parameter corresponding to the area surrounded by the dashed line is suitable for phase pure copper sulfide production. Parameters outside this region produce different phases of copper nanoparticles.

An enclosed flame spray reactor was utilized to create a reducing environment by controlling the supplied fuel‐to‐oxygen ratio in the flame, which created a suitable reaction environment for metal sulfide production. Precursor‐solvent interaction and various process parameters such as precursor feed rate, dispersion oxygen flow rate, co‐flow rate, and metal‐to‐sulfur ratio are the fundamental determining factors for particle nucleation and growth mechanism. Metal conjugate bonds between Cu and π bond of thiophene exist in the miscible liquid precursor solution, which indicates that metal sulfide formation happens only in the gas phase. A sufficiently reducing environment for copper sulfide formation can be ensured by maintaining a fuel‐to‐oxygen ratio of 1.5 or higher (**Figure** **7**). A fuel‐to‐oxygen ratio lower than 1.5 leads to insufficient reducing conditions, which introduces oxide impurity to the particles, and a fuel‐to‐oxygen ratio higher than 2 leads to incomplete combustion, which also introduces unburned carbon and sulfur content to the particles. Unlike metal oxides, the increase of dispersion oxygen flow increases the particle diameter due to the high temperature produced by enhanced combustion.

A minimum Cu to S ratio of 1:5 is necessary (Figure 7) in the solution to ensure a sufficient concentration of sulfur in the gas phase and a Cu to S ratio of more than 1:30 introduced un‐bonded sulfur content in the particles. Increasing the Cu to S ratio decreases the particle diameter due to an increased nucleation of the particle. Particle primary diameter increases with increasing feed rate and feed rate equal to or more than 7 mL min^−1^ leads to exiting aerosol temperature ≈520 °C that converts the copper sulfide into copper sulfate hydroxide hydrate and copper oxide phase. A low co‐flow rate of 100 L min^−1^ is insufficient to cool down the stream, and at that low co‐flow the temperature of the aerosol stream is so high that it converts the metal sulfide into a hydrated sulfate phase. As the co‐flow rate increases, the particle diameter decreases because the particle resides in a low‐temperature flame for higher co‐flow. With this knowledge, the parameters can be optimized to synthesize tailored metal sulfide nanoparticles with certain phase composition, crystallinity, and particle diameter to bring metal sulfide into a new application field. This study will also help to perform in situ coating, doping, mixing, and functionalizing the metal sulfide nanoparticle in a reducing environment. Furthermore, as‐prepared copper sulfide shows a narrower bandgap and higher charge carrier mobility compared to its oxide counterpart, which indicates its potential application in the field of gas sensors, photo‐catalysts, and solar cell material.

## 5 Experimental Section

### Precursor Preparation

To prepare copper sulfide nanoparticles, solid Cu 2‐ethylhexanoate (purchased from Sigma–Aldrich, solid, 17–19% Cu) was dissolved in thiophene (purchased from Sigma–Aldrich, product no. T31801, 99.9% pure) and tetrahydrofuran (Sigma–Aldrich product, product no. 178 810, 99.9% pure). Solutions with different Cu to S ratios (1:30, 1:20, 1:10, 1:5) were prepared to adjust the volume of thiophene while maintaining Cu concentration 0.25 mol L^−1^.

### Nanoparticle Synthesis

A custom‐made flame spray pyrolysis (FSP) reactor was employed to produce copper sulfide nanoparticles in a controlled atmosphere under fuel‐rich conditions. In this process, a liquid precursor was introduced into the spray nozzle via a 100 mL syringe (Hamilton 1000) derived by a Legato 210 syringe pump at flow rates of 3, 5, and 7 mL min^−1^. The precursor was then dispersed into a spray by adjusting the oxygen flow rate to accommodate different fuel‐to‐oxygen ratios while maintaining a constant pressure drop of 1.5 bar. The spray was ignited using a premixed methane‐oxygen flame, consisting of CH_4_ at a flow rate of 1.2 L min^−1^ (2.5, with a purity of 99.5 vol%, Westfalen) and O_2_ at a flow rate of 2.2 L min^−1^(3.5, with a purity of 99.95 vol %, Westfalen). This flame was supplied through an annular gap surrounding the spray. To enclose the flame, a quartz tube (ProQuartz, SK2533 Rohr) with an inner diameter of 100 mm and a length of 50 cm was used. This tube was positioned on a stainless‐steel porous ground plate, and the top part of the tube was inserted into a stainless‐steel filter housing. This setup was designed to shield the flame from direct contact with the surrounding air, ensuring controlled conditions for the combustion process. A co‐flow of nitrogen gas (5.0 and purity of 99.999 vol%, Westfalen) was supplied through the porous ground plate to transport the particles out of the quartz tube. All gases were precisely controlled using calibrated Bronkhorst mass flow controllers integrated with a LabView program. Nanoparticles were collected from the flame by placing a glass microfiber filter in the upper part of the filter housing downstream of the flame, while a vacuum pump (SECO SV 1025) was used to draw the particles from the gas flow and deposit them onto the filter. The particles were scratched off from the filter and subsequently filtered through a 250 µm mesh sieve. The collected particles were then stored in a paraffin foil‐sealed jar to prevent any kind of contamination, such as moisture adsorption.

### X‐Ray Diffraction (XRD) Measurements

The copper sulfide samples were loaded on top of a Si holder in a Bruker D8 Discover instrument in a Bragg–Brentano setup with Cu‐Kα_1_ (λ = 0.15406 nm) radiation. The instrument had 0.26° fixed divergence, a radius of 250 nm, and a LynxEye XeT detector with a total acceptance angle of ≈3.3° in 2ϴ‐scale, distributed across 192 channels with 0.015625° channel width and a continuous scan was conducted within the range of 15–100° in 2ϴ‐scale for 5h 59 min for each sample. The integration step width during scanning was set at 0.0118613° in 2ϴ‐scale. The purity and composition of the copper sulfide nanoparticles were determined using Rietveld refinements of the XRD patterns with the BRASS program. Scale factor, background, unit cell parameter, Gaussian and Lorentzian peak width parameter were refined using crystal structures from the Inorganic Crystal Structure Database (ICSD 41 142 and ICSD 41 911). The quality of the Rietveld refinement was assessed using the numerical profile agreement factors (*R_wp_*, *R_p_*, *R’_p_*, *R_Bragg_*).^[^ ^65^ ^]^ The quality of the Rietveld‐refined patterns was assessed through visual inspection of the fit between observed and calculated data, evaluation of numerical profile agreement factors (R_wp_, R_p_, R'_p_), and, in particular, the phase‐specific agreement factors (R_Bragg_).^[^ ^66^ ^]^

### Surface Area Measurements

The specific surface area of the samples was determined using an autosorb gas sorption system (Quantachrome NOVA 4000e) with the Brunauer–Emmelt–Teller (BET) method. The powder was placed in test cells under vacuum conditions and degassed at 50 °C for 24 h. The BET isotherm measurements were performed using nitrogen gas as adsorbent at 77 K and 5 points were measured in a relative pressure (P/Po) over the range of 0.09–0.30. The average equivalent particle diameter was calculated assuming a spherical shape of the particle using the equation *d_BET_* = 6/(ρ_p_ x *SSA*), where *d_BET_* is the average diameter of a monodispersed particle calculated from the measured specific surface area (SSA) of the powder and density of the particle (ρ_p_).

### Transmission Electron Microscopy (TEM) Measurements

The TEM samples were prepared by dispersing ≈(1–2) mg powder in a 10 mL ethanol solution and then sonicated for 10 min. A few drops of the resulting solution were transferred to Plano carbon‐coated TEM Ni‐grids and dried at room temperature. Low‐resolution TEM (LRTEM) images, high‐resolution TEM (HRTEM) images, and selected area electron diffraction (SAED) images were obtained using an image‐corrected FEI Titan 80–300 ST instrument. A Thermo Fischer Spectra 300 instrument equipped with an energy‐dispersive X‐ray (EDX) detector was used for elemental mapping.

### Fourier Transform Infrared Spectroscopy (FTIR) and UV–Vis

The FTIR spectra of the prepared solution were obtained utilizing an Agilent Cary 630 instrument equipped with a ZnSe crystal using the attenuated total reflection (ATR) method. A high signal‐to‐noise ratio was ensured by averaging 16 scans for every sample. The spectra were captured within the wavenumber range of 650 to 4000 cm^−1^ with a nominal resolution of 2 cm^−1^.

UV–vis spectra were measured using a Shimadzu UV‐2600 instrument in an absorption mode with a medium scan speed from 300 to 700 nm wavelength utilizing a direct detector unit. Reference cuvette was used to cancel undesired background signals from the measured cuvette signal data. The concentration of the solution used in the cuvette was maintained at 10^−3^ mol L^−1^ to ensure absorption lower than 1.0.

### Bandgap Measurement

UV–vis reflection spectra were captured using a photo spectrometer (UV‐2600, Shimadzu) equipped with an integrating sphere containing BaSO_4_ as a background material. A Kubelka‐Munk transformation was conducted with the acquired data to determine the bandgap of the nanoparticles.

### Electrode Fabrication

After the synthesis of nanoparticles using FSP, the particles were transferred from the glass fiber filter unit to a substrate using low‐pressure roll‐to‐roll lamination (Hot Roll Laminator HL‐101, Chem instruments). The substrate was made of conductive FTO glass slides which were compressed during lamination at a pressure of 3.2 MPa. To prevent electrolyte contact with the substrates in electrochemical impedance measurements, porous layers were impregnated with a 1,6‐hexanediol diacrylate monomer solution containing 1 wt% UV photoinitiator. Subsequently, the infiltrated layers were illuminated with a UV light source for 90 s in a nitrogen‐inert atmosphere to polymerize the monomer. The outermost polymer coating was removed by grinding against silicon carbide foil at 60 rpm rotation speed using the Tegramin‐25 (Struers) machine at 5N force for 15 min.

### Electrochemical Characterization

The electrochemical impedance spectroscopy (EIS) technique was conducted to characterize the electrochemical properties of the as‐prepared nanoparticle layers. A three‐electrode electrochemical cell was used which consists of the porous nanoparticle working electrode (8 cm^2^), a Pt‐coated grid as a counter electrode, and Ag/AgCl (3m KCl, SI Analytics) as a reference electrode. The impedance spectra were recorded on a potentiostat (VMP 300, Biologic with the Software EC‐Lab), and 0.5m Na_2_SO_4_ (PH 6.7) was used as an electrolyte.

## Conflict of Interest

The authors declare no conflict of interest.

## Data Availability

The data that support the findings of this study are available from the corresponding author upon reasonable request.
